# Aspergillus Coinfection in a Hydatid Cyst Cavity of Lung in an Immunocompetent Host: A Case Report and Review of Literature

**DOI:** 10.1155/2023/6975041

**Published:** 2023-07-14

**Authors:** Aayush Adhikari, Surendra Khanal, Sagar Rana Magar, Srijana Thapa, Srijana Khati, Rajan Lamichhane, Kundan Marasini

**Affiliations:** ^1^Manang District Hospital, Chame, Nepal; ^2^Department of Internal Medicine, Piedmont Athens Regional, Athens, Georgia, USA; ^3^Maharajgunj Medical Campus, Institute of Medicine, Kathmandu, Nepal; ^4^Manmohan Cardiothoracic Vascular and Transplant Center, Kathmandu, Nepal; ^5^Department of Pathology, Tribhuvan University Teaching Hospital, Kathmandu, Nepal; ^6^Department of Radiology, Tribhuvan University Teaching Hospital, Kathmandu, Nepal

## Abstract

Aspergilloma (a saprophytic infection) typically colonizes lung cavities due to underlying diseases such as tuberculosis, bronchiectasis, cavitary lung cancer, sarcoidosis, and pulmonary infarctions. Rarely, aspergilloma has been noted within a hydatid cyst. Even if this was the case, it is more common to find the coexistence of aspergilloma and pulmonary echinococcal cysts in immunocompromised individuals. It is, however, very uncommon to find this coinfection in normal immune status individuals. Here, we report on the successfully treated case of a 30-year-old immunocompetent female from Western Nepal with histologically proven coinfection by these two pathogens. She had a prolonged history of exposure to domesticated dogs. She suffered from hemoptysis from time to time for 3 years with increased frequency in the last 30 days. She was misdiagnosed clinically during a past medical visit at a local health center. Her computed tomography (CT) scans showed well-defined nonenhancing cystic lesions in the anterior basal segment of the right lower lobe adjacent to the major fissure. She underwent enucleation of the cyst via right posterolateral thoracotomy. On further histopathological evaluation, laminated membranes of the ectocyst along with fungal elements were found, and periodic acid-Schiff (PAS) staining revealed Aspergillus in the form of septate hyphae and acute angle branching. Owing to patient's economic constraints and unavailability in our center, DNA testing and molecular characterization could not be performed which further highlights the essence of diagnosing and managing such cases in resource poor settings. Eventually, we reviewed 12 confirmed cases of this coinfection in immunocompetent individuals during a period of 7 years (2015–2022) comparing them to a systematic review of 22 confirmed cases of the same coinfection from 1995 to 2014.

## 1. Introduction

Echinococcosis, a worldwide zoonosis and one of the 17 neglected tropical diseases enlisted by the World Health Organization (WHO) is a health problem of concern in communities where animal husbandry and traditional agricultural activities are part of living. Dogs, the definitive hosts, transmit infection to humans (aberrant intermediate hosts) through the faecal-oral route, as tapeworms pass proglottids and eggs in dogs' faeces. There are two major species that are of particular medical and public health importance: *Echinococcus granulosus* and *Echinococcus multilocularis*, which cause cystic echinococcosis (CE) and alveolar echinococcosis, respectively [1–3].

Hydatid cysts of *Echinococcus granulosus* develop mostly in the liver and lungs of humans. Most patients remain asymptomatic in the early phases of infection. Symptoms follow as the cysts become larger or complicated with varying manifestations based on size, location, compression of nearby structures, and condition of the cystic structures [4, 5].

Pulmonary hydatidosis coinfected with cryptococcosis, aspergilloma, and saprophytic fungus has been reported in the literature [6–8]. *Aspergillus* species can generally colonize pre-existing lung cavities caused by tuberculosis, sarcoidosis or bronchiectasis; however, it is extremely rare to find an aspergilloma developing in pulmonary CE. Infection by Aspergillus can occur in immunocompromised patients but coinfection of aspergilloma with an unruptured pulmonary hydatid cyst in immunocompetent individuals is very unlikely [9].

The diagnosis of hydatid disease is based on imaging techniques (CT, MRI, ultrasound, and radiography) which identify the space-occupying cysts or lesions caused by developing, dying, or dead metacestodes of *Echinococcus* species. Surgery remains the mainstay of treatment, aiming to preserve the lung parenchyma where possible with parenchymal resections reserved as last-resort options [9, 10]. The coinfection by these pathogens can result in massive hemoptysis and even dreadful invasive disease for which early diagnosis and treatment are crucial [8].

We hereby report the successful treatment of a coexistent pulmonary hydatid cyst and aspergilloma in a previously misdiagnosed, immunocompetent young female.

## 2. Case Report

A 30-year-old female farmer and nonsmoker with chief complaints of hemoptysis from time to time for 3 years with increased amount and frequency for the last 30 days was admitted to Manmohan Cardiothoracic Vascular and Transplant Center, Kathmandu, Nepal. At the time of admission, she complained of cough with associated hemoptysis and chest pain.

She was in her usual state of health 3 years back when she developed coughing up blood with a frequency of 1-2 episodes a month. With these symptoms, 7 months later, she visited a nearby medical center where she was diagnosed and treated for pneumonia. The symptoms persisted with occasional episodes of coughing up blood for which no medical intervention was sought. Around a year back from now, she noticed the presence of whitish pieces mixed with blood for 2 episodes while coughing up, which was associated with a sour/bitter taste in her mouth. With the persistence of her symptoms of coughing up blood, a few months later she visited a tuberculosis center near her village where a CT scan demonstrated lesion on her lungs, and she was diagnosed of having a hydatid cyst in her lungs. From there, she was referred to our center for further management.

She denied a history of fever, shortness of breath, night sweats, significant weight loss, contact with TB, history of travel, family history of malignancy, the use of immunosuppressive drugs, and similar illnesses in family members. There was no history of pre-existing pulmonary diseases, extrapulmonary symptoms, and known previous medical problems in the patient. Tracing her history, her home was near a jungle, and including hers, nearly all homes had domesticated dogs. She gave a history of prolonged exposure to domesticated dogs. She was a nonsmoker, nonalcoholic, and consumed a mixed diet.

In our center, her vital signs showed normality with a blood pressure of 118/70 mm·Hg, a pulse rate of 80 beats/minute, a respiratory rate of 18 breaths/minute, an oxygen saturation of 97% in room air, and an axial body temperature of 98 degrees Fahrenheit. Routine laboratory examination showed no abnormality with hemoglobin of 12 g/dl and there was no eosinophilia on peripheral blood examination. Total and differential leukocyte counts were within normal limits. Serum electrolytes, liver function tests, renal function tests, and other systemic examinations were unremarkable. Sputum smears were negative for acid-fast bacilli. She was nonreactive for Hepatitis B and HIV antigens. Physical findings were confined to the chest with decreased breath sound on the right hemithorax. The laboratory parameters of our patient at the time of admission are tabulated in Table 1.

**Figure 1:**
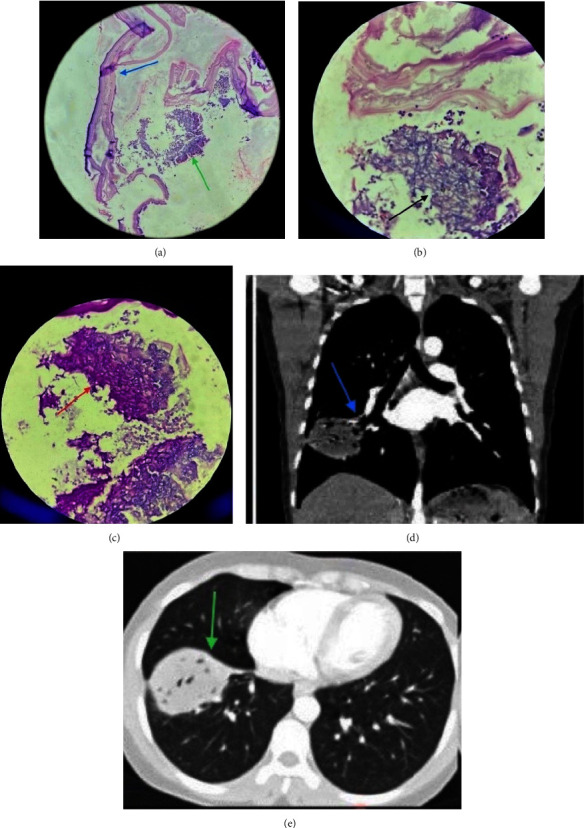
(a) Low-power view (H&E *∗* 100) showing laminated membrane of ectocyst (blue arrow) along with fungal elements (green arrow). (b) High-power view (H&E *∗* 400) showing septate hyphae with acute angle branching (black arrow) supporting evidence of Aspergillus in hydatid cyst cavity. (c) PAS stain showing Aspergillus hyphae with acute angle branching and thin septa (red arrow). (d) CECT chest (coronal view) showing segmental bronchus within the cyst with possibility of communication with the cyst (blue arrow). There is segmental collapse and upward pulling of the right hemidiaphragm. No obvious pleural or chest wall communication seen. (e) CECT chest (axial view) showing well-defined nonenhancing cystic lesion in anterior basal segment of right lower lobe adjacent to major fissure (green arrow). Multiple air foci are seen within it.

Her chest CT upon presentation showed well-defined nonenhancing cystic lesion in the anterior basal segment of the right lower lobe adjacent to a major fissure. Multiple air foci were seen within it. There was a segmental bronchus seen within the cyst with the possibility of communication with the cyst. It was causing segmental collapse with the upward pull of the right hemidiaphragm. No obvious pleural or chest wall communication was seen (Figures 1 (d) and (e)). There was no associated liver lesion on abdominal ultrasonography.

The surgery was performed via the right posterolateral thoracotomy from the 5^th^ intercostal space. Intraoperatively, the cyst was approximately of size 7.5 *∗* 5.5 cm in the superior segment of the right lower lobe. Four bronchial communications were identified. A cystectomy was done, and at least 4 cystobronchiole communications were noted which were individually closed with nonabsorbable sutures, and then capitonnage was also performed. The excised cyst was sent for histopathological examination. What was exceptional was the presence of a laminated membrane of the ectocyst along with fungal elements (Aspergillus) in the form of septate hyphae with acute angle branching in hematoxylin and eosin stain (H&E) (Figures 1 (a) and (b)). To further confirm this finding, Periodic acid-Schiff (PAS) staining was done which also showed acute angle branching with thin septa (Figure 1 (c)). This led us to the final diagnosis of coinfection by *Echinococcus* and Aspergillus. She has been on oral albendazole since the first postoperative day at a dose of 10–15 mg/kg/day in divided doses. Her stay after surgery was uneventful and she was discharged on 3 postoperative days. She had been on routine follow-up.

## 3. Discussion

Hydatidosis has a worldwide distribution as one of the most common zoonotic diseases between humans and animals. It is particularly endemic in the Middle East, the Mediterranean, Southern Europe, Latin America, East Africa, Australia, and New Zealand. The major determinants of hydatidosis include conventional animal husbandry, nomadic/seminomadic ways of life and food behavior including consumption of uncooked vegetables. Stray dogs ingesting the infected viscera of sheep or goats and contaminating the vegetables in open fields significantly contribute to the seropositivity of CE [1, 9].

The liver followed by the lungs are the most commonly involved organs representing about 60–70% and 20% of all cases, respectively [11]. The coinfection of hydatid cyst with viral and bacterial infections has been reported in the literature. Aspergilloma (a saprophytic fungal infection) has generally been noted in lung cavities including pre-existing cavities caused by tuberculosis, sarcoidosis, and bronchiectasis. Also, Aspergilloma and hydatid cyst in isolation are known to occur in lungs; however, its coexistence in pulmonary hydatid cyst cavities is extremely rare and reported only occasionally in the literature. Aspergilloma can develop in a pulmonary hydatid cyst after the onset of surgery or years thereafter while our patient had concurrent Aspergillus and *Echinococcus* infections [8, 9, 12]. Immunocompromised patients are particularly more prone to Aspergillus infections and there are scarce cases of coexistence of Aspergillus and pulmonary echinococcosis in immunocompetent individuals [6, 13, 14]. Koçer et al. while re-evaluating 100 archival cases of hydatid cyst in a retrospective study of coexistence of hydatid cyst and aspergillosis found that only two immunocompetent cases had coinfection of hydatid cyst and aspergilloma [15].

Symptomatology depends on the size and location of hydatid cysts and initially the course is mostly asymptomatic. The majority of symptoms in pulmonary hydatid disease are caused by the mass effect where cyst volume compromises nearby structures. Complications due to pulmonary hydatid cysts depend on size, site, and whether the cyst is intact or not. Intact cysts usually lead to compressive symptoms. Depending on bronchial and/or pleural irritation, chest pain and coughing may develop. Atelectasis due to parenchymal compression and the resultant pneumonia and hemoptysis due to vessel erosion can also develop as a complication of intact cysts. In the case of ruptured cysts, asphyxia, hemoptysis, anaphylactic shock, respiratory failure, pneumonia, and bronchiectasis are the major complications [16]. In a 5 years' experience study at a tertiary lung center in Iran, 234 patients with pulmonary hydatidosis were evaluated. The most common symptoms included cough (59.8%), dyspnea (31.1%), hemoptysis (26%), chest pain (14.1%), and abdominal and flank pain (2.9%) [17]. Aspergillus has a tendency to invade blood vessels, and hence hemoptysis is a common symptom of pulmonary aspergillosis. Also, hemoptysis occurs as a result of reactive vascular granulation tissue, making it the most common presentation in pulmonary aspergilloma [18, 19].

Combined radiographic and immunodiagnostic techniques can be used as a reliable noninvasive modality for confirming the diagnosis of pulmonary echinococcosis. Apart from conventional radiography, imaging modalities including computed tomography, magnetic resonance imaging, and ultrasonography can readily identify deep-seated lesions in all organs as well as the extent and condition of avascular fluid-filled cysts [20]. Ultrasonography as an easily available modality should be performed to identify accompanying liver involvement in patients with pulmonary echinococcosis. Concomitant cysts in the liver and lungs are found in about 15% of cases which can further aid in diagnosis [21, 22].

Immunological testing owing to its lower sensitivity and specificity plays a subsidiary role in diagnosis. The World Health Organization/World Organization for Animal Health has put forward a sequential screening and confirmatory test model. Enzyme-linked immunosorbent assays (ELISA), indirect hemagglutination antibody tests (IHAT), latex agglutination (LAT), immunofluorescence antibody tests (IFAT), and immunoelectrophoresis (IEP) can be applied for primary screening. In reactive primary screening groups, the inaccuracy due to cross-reactivity with other parasitic conditions including alveolar echinococcosis, cysticercosis, fascioliasis, and filariasis can be surmounted by a confirmatory test which includes immunoblot assays to test for reactivity with *E. granulosus* antigen subunits, recognition of specific IgG subclasses (i.e., IgG1 and/or IgG4), and arc 5 precipitation testing. However, pulmonary cysts are less likely to elicit an immune response than hepatic cysts with a seropositivity of around 65% in pulmonary cases and 80% to 94% in hepatic cases [23, 24]. Eosinophilia can be found in around 10–30% of patients with hydatid disease. In cases where cyst ruptures and in endemic areas, eosinophilia is likely to be more pronounced [25].

Imaging findings in pulmonary hydatidosis vary depending on the presence of complications which are classified as uncomplicated or complicated (contained rupture, complete rupture, and superinfection). The characteristics of an uncomplicated hydatid cyst are a well-defined homogeneous lesion with varying smooth wall thickness. CT density measurement corresponds with fluid content at low HU values. The central cysts tend to be round while the peripheral ones are oval to polycyclic, and it is rare to see daughter cysts and calcifications in pulmonary hydatid cysts. In around 47.5% of cases, pulmonary hydatid cyst may rupture due to degeneration of cyst membranes, the causation of which is multifactorial. The ruptured hydatid cysts show different radiological signs and may show higher HU due to mucus, infection, or hemorrhagic content [26].

In CT scans of coexistent aspergilloma and hydatid cysts, globules of gas within the hyphal ball can be revealed, either loose or adherent to the cavity wall by granulation tissue. The mobility of the fungal ball within the cavity can be elucidated when the cavity is not fully occupied by the fungal mass, and this is called “monod sign.” Another sign called the air crescent sign is also an important specific sign in aspergilloma. However, radiographically, the sensitivity and specificity of identifying Aspergillus colonies trapped in hydatid cysts are unclear, and both of these signs can be seen in hydatid cyst, pulmonary tuberculosis, pulmonary abscess, pneumocystis pneumonia, and bronchogenic carcinoma [27, 28]. In cases where degeneration of the cyst or infection eludes the radiographic and serological diagnosis, pathologic evaluation or positive culture helps in confirming the diagnosis [29]. Moreover, the concomitant infection with Aspergillus can also be visualized in histology, as in our case. The presence of cyst-like lesions in a person from an endemic region is a supportive factor in diagnosing cystic echinococcosis. However, it must be distinguished from benign cysts, mycoses, cavitary tuberculosis, abscesses, and benign or malignant neoplasms [24].

Surgery and total excision are the mainstays of conservative treatment for pulmonary hydatid cysts. With the chances of recurrences being too low, a parenchyma preserving approach is best recommended. However, segmentectomy and even lobectomy may be inevitable sometimes. Commonly, Barrett/Posadas' technique (cystotomy and closure of bronchopleural fistulas with or without capitonnage) is used. There are various viewpoints regarding capitonnage and one notion of surgeons is that good results can still be obtained as the residual cavity will obliterate on its own. When residual cavities are present after cystectomy, long-term follow-up is recommended [10, 30, 31].

We identified and reviewed case reports of concomitant hydatid cyst and aspergilloma, which presented similarly to this case. The detailed comparison of those cases with ours is tabulated here in Table 2.

Aliyali et al. in their case report and systematic review of concomitant pulmonary hydatid cyst and aspergilloma in 2015 reported hemoptysis as the most common symptom (75%). In the same study, 76% of cases underwent standard lobectomy and the remaining 24% had one of thoracotomy, pneumonectomy, and cystectomy. Apart from their case, 52% of cases were treated with azole agents and 29% received anthelmintic therapy [9].

We found 11 published cases of this coinfection after 2015 during the literature search. All individual cases including ours were immunocompetent, and the major presenting complaint was hemoptysis (81.8%) in 9 of 11 cases including ours which was followed by cough, chest pain, or dyspnea. The mean age of patients including our case was 38.9 years with 58% being male. Antifungal agents or anthelminthic agents were not used in our case preoperatively. 4 cases received antifungal or anthelminthic therapy before surgery while one case was treated medically only [13, 34–36, 39]. In our review of 11 published cases, standard lobectomy was performed in 5 cases, 5 cases underwent thoracotomy or cystectomy with/without capitonnage, and 1 case was treated medically only.

A cross-sectional survey assessing the knowledge, attitude, and practice (KAP) of 180 surgically treated cases of hydatid cyst in southern Iran found poor knowledge and awareness of participants towards the disease. Higher age and lower education level were associated with lower knowledge and urban living location was associated with a more positive attitude towards the disease. These results highlight the role of awareness programs through health education to prevent and control this neglected parasitic infection [41].

## 4. Conclusion

In the present report, we have presented a previously misdiagnosed case of pulmonary hydatid cyst and Aspergillus coinfection in an immunocompetent young female. She had a 3-year-long history of intermittent hemoptysis and was misdiagnosed during that period of health visit, and the increased severity of hemoptysis in the last 30 days led her to seek medical intervention in our center. A definitive diagnosis was established after a CT scan and histopathological evaluation of samples, and she was successfully treated with surgery. It is crucial to bear in mind the possibility of secondary infection of a hydatid cyst cavity by saprophytic fungus for better management of pulmonary hydatid cysts and avoidance of complications, even in immunocompetent individuals. Surgical removal of the cyst is mostly curative in immunocompetent individuals with this coinfection. In immunocompromised individuals, the course of this coinfection can be life-threatening and follow-up with serological tests and imaging and prophylactic chemotherapy targeting aspergillosis may help in reducing further complications.

## Figures and Tables

**Table 1:** Laboratory parameters at the time of admission.

Blood parameters	Values	Reference ranges
Total leukocyte count	6,400/mm^3^	4–11 *∗* 10^3^/mm^3^
Platelets	1,82,200/mm^3^	150–350 *∗* 10^3^/mm^3^
Hemoglobin	12 g/dL	Female (11.5–16.5 g/dL)
PT/INR	14/1.0	PT: 10.7–15.3 seconds INR: 1.0–1.1
Random blood glucose	97 mg/dL	Fasting (65–104 mg/dL)
Na/K	137/3.6 mEq/L	Na: 135–145 mEq/L, K: 3.6–5 mEq/L
Total bilirubin	0.4 mg/dL	0.18–0.94 mg/dL
Direct bilirubin	0.2 mg/dL	0–0.2 mg/dL
ALT/AST	17/22 U/L	ALT: 10–50 U/L AST: 10–45 U/L
ALP	68 U/L	40–125 U/L
Albumin	4.2 g/dL	3.5–5 g/dL

**Table 2:** Comparison of published cases of concomitant pulmonary hydatid cyst and aspergilloma.

Case references	Study types	Age (years)/sex	Symptom on presentation/immunocompetency or underlying disease	CT findings	Treatment
Present case	Case report and literature review	30/F	Chest pain, cough, and hemoptysis/immunocompetent	Well-defined nonenhancing cystic lesion in the anterior basal segment of the right lower lobe adjacent to major fissure	Right thoracotomy, cystectomy, and capitonnage

Aliyali et al. [9]	Case report and systematic review, total cases = 22	Mean age = 40.8/70% M	75% had a symptom of hemoptysis/all immunocompetent	Cavitary lesion (17/22), opacified lesion (2/22), ruptured hydatid cyst (1/22), residual cystectomy cavity (1/22), no cavity (1/22)	Standard lobectomy procedures (76%), thoracotomy, pneumonectomy, and cystectomy (24%)

Zareshahrabadi et al. [8]	Case report	42/M	NA/immunocompetent	Circumscribed cystic lesion in the superior and inferior segment of the lower lobes of right and left lungs	Radical surgery (lobectomy)

Aala et al. [13]	Case report	34/F	Chest pain, dyspnea with nonproductive cough/immunocompetent (h/o hyperthyroidism)	Subpleural consolidation with small cavitation and pleural effusion, thickening and enhancement in left lower lobe	Albendazole and oral itraconazole and thoracotomy
				Liver-cystic lesion in caudate lobe of liver (33 mm)	

Chopra and Katoch [32]	Case report	43/M	Productive cough, low-grade intermittent fever, and right-sided nonpleuritic, nonanginal chest pain, and streaky hemoptysis/immunocompetent	Thick-walled cavity in the superior segment of the right lower lobe with an intracavitary mass	Lobectomy of both right middle and lower lobes

Rezaei et al. [33]	Case report	37/F	Cough, chest pain, and dyspnea/immunocompetent	Inflammatory mass clinging to the chest wall with cavity in the anterior segment of the right upper lobe	Right posterolateral thoracotomy, cystectomy, and capitonnage

Sharma et al. [34]	Case report	20/M	Episodic cough with mucoid expectoration which was associated with streaky hemoptysis with episodic breathlessness/immunocompetent	Complex lesion of 3.8 × 2.9 cm in size in right upper lobe with eccentric air crescent	Tablet albendazole 10 mg/kg/day with liver function test monitoring for total duration of 12 months

Rao et al. [35]	Case report	55/M	Hemoptysis/diabetic	Well-defined lesion in left lower lobe of lung with mediastinal lymphadenopathy	Antifungal, anthelminthics, postero-lateral thoracotomy

Singh et al. [36]	Case report	43/M	Mild weakness, cough, dyspnea, hemoptysis, and chest pain/history of minor thalassemia	Large cavitary lesion (5 × 6 × 6 cm) involving left lower lobe of lung	Surgical excision of the cyst after a course of anthelmintic treatment

Aboksari and Safavi [37]	Case report	10/M	Chest pain, low-grade fever, nonproductive cough, and malaise/immunocompetent	Complicated, ruptured cyst containing air	Total cystectomy and capitonnage
Goyal et al. [38]	Case report	45/M	Breathlessness, hemoptysis/immunocompetent	Well-defined peripherally enhancing thick-walled cystic lesion in the middle lobe of the right lung	Middle lobectomy with video-assisted thoracoscopic surgery (VATS)

Nayak et al. [39]	Case report	36/F	Dyspnea, chest pain, cough with sputum production, intermittent hemoptysis/immunocompetent	Two well-defined hypodense mildly enhancing lesions with smooth margins involving posterior basal and lateral basal segment of the lower lobe of the left lung	Antifungal and anthelmintic agents
					After 6 months-left lower lobectomy

Salazar et al. [40]	Case report	72/F	Productive cough and recurrent episodes of massive hemoptysis, dyspnea/immunocompetent	Extensive cavitary lesion with heterogeneous content in segment 3 of left upper lobe	Surgical resection of lesion-multiple segmentectomy

## Data Availability

All the necessary data and information are within the article.
